# Progress in building a longitudinal framework of linked reference data for England and Wales.

**DOI:** 10.23889/ijpds.v7i3.1875

**Published:** 2022-08-25

**Authors:** Kostas Loukas, Paul Groom

**Affiliations:** 1 Office for National Statistics

The Office for National Statistics (ONS) is working to transform its statistics, moving away from a system that is based on a ten-yearly Census and supporting surveys, to one that uses all available data to produce the highest quality statistics at any given time. This transformation requires that analysts be supplied with new data, new methods, and a new platform.

We are developing a longitudinal framework to structure and integrate administrative data; indexing data relating to people, business, location and classifications. The core reference data indexes aim to maximise the use and value of administrative data for statistical purposes within ONS where data standards, security and consistency are designed in from the start. Assigning index ID’s to data allows personally identifiable information to be removed without impacting the usability of the data. The framework enables analysis on data with links between people, businesses and locations.

The Demographic Index (DI) clusters together administrative records of a population entity (person) and assigns them a Unique ID. The administrative data sources are selected based on their quality and their population coverage. As there is no unique person identifier available across these sources both deterministic and probabilistic linking methods are used to build the index. The design of the index and how it is updated and maintained over time must meet ONS statistical research needs whilst also adhering to data warehousing standards and keeping personal information safe. The result is an anonymised database of longitudinally linked administrative data that is used in the production of population statistics and other statistical projects.

The creation of the DI has many challenges and further development will focus on how we measure and communicate quality to the users, investigating methods to reconcile attributes that are not align across sources and managing the linkage error as the DI is updated with more data.

